# Polygenic risk of Social-isolation and its influence on social behavior, psychosis, depression and autism spectrum disorder

**DOI:** 10.21203/rs.3.rs-2583059/v1

**Published:** 2023-03-02

**Authors:** Adam Socrates, Niamh Mullins, Ruben Gur, Raquel Gur, Eli Stahl, Paul O’Reilly, Abraham Reichenberg, Hannah Jones, Stan Zammit, Eva Velthorst

**Affiliations:** Icahn School of Medicine at Mount Sinai; Icahn School of Medicine; University of Pennsylvania; Icahn School of Medicine at Mount Sinai; Icahn School of Medicine at Mount Sinai; Icahn School of Medicine at Mount Sinai; University of Bristol; University of Bristol; Icahn School of Medicine at Mount Sinai

## Abstract

Social-isolation has been linked to a range of psychiatric issues, but the behavioral component that drives it is not well understood. Here, a GWAS is carried out to identify genetic variants which contribute to Social-isolation behaviors in up to 449,609 participants from the UK Biobank. 17 loci were identified at genome-wide significance, contributing to a 4% SNP heritability estimate. Using the Social-isolation GWAS, polygenic risk scores (PRS) were derived in ALSPAC, an independent, developmental cohort, and used to test for association with friendship quality. At age 18, friendship scores were associated with the Social-isolation PRS, demonstrating that the genetic factors are able to predict related social traits. LD score regression using the GWAS demonstrated genetic correlation with autism spectrum disorder, schizophrenia, and major depressive disorder. However, no evidence of causality was found using a conservative Mendelian randomization approach other than that of autism spectrum disorder on Social-isolation. Our results show that Social-isolation has a small heritable component which may drive those behaviors which is associated genetically with other social traits such as friendship satisfaction as well as psychiatric disorders.

## Introduction

Social contact is essential for surviving and thriving in human societies^1^. As such, having limited contact with other people, or Social-isolation, can have detrimental effects on both physical and mental health. There is evidence that lack of social contact is associated with schizophrenia^2,3^, autism spectrum disorder^3^, and depression^4^, as well as with medical conditions such as cardiovascular disease^1,5^ and diabetes^6^. Longitudinal studies indicate that Social-isolation can predate mental issues and have a strong causal effect on poor mental health outcomes^4,7,8^. These issues have been acutely brought to light in the context of the Covid-19 pandemic, in which forced social isolation has had a substantial negative effect on mental health^9^. Social-isolation has been found to be strongly associated with the development of psychosis, and it has been hypothesized that this contribution may be due to negative, delusional or paranoid thoughts not being tested in reality and therefore corrected in social interactions^10,11^

Despite the impact of Social-isolation on mental and physical health, it remains one of the least studied factors in psychiatric disorders, limiting understanding of aetiology and causality with regards to psychiatric disorders ^12,13,14,15^. Associations between genetics and traits related to social contact such as feelings of loneliness (feelings of distress or discomfort from being alone) and sociability (the ability to connect and socialize with others) have been noted^16^. However, the existence and influence of an exclusive genetic predisposition towards Social-isolation *behaviors*, i.e., action that leads to isolation, is yet to be established. Consequently, there is a fundamental gap in our knowledge about the extent to which Social-isolation may represent a *causal and independent* risk for poor mental and physical health instead of being merely a direct *consequence* of other (clinical) symptomatology, for example due to stress or feelings of paranoia.

Twin studies have demonstrated that there is a similar genetic influence on both social isolation (40%) and loneliness (38%), but that they are only moderately genetically correlated, reflecting partially distinct constructs^17^. However, to our knowledge no prior study has carried out a genome-wide association study (GWAS) to elucidate the polygenic component of the purely behavioral aspects of Social-isolation, as distinct feelings relating to social behavior such as loneliness. This is pertinent, as these behaviors could provide modifiable early intervention targets if found to be on the causal pathway between inherited genetic variation and psychiatric disorders^18^.

In order to better understand the genetic factors that influence Social-isolation, the present study (1) conducted a novel GWAS for Social-isolation behavior in the UK Biobank cohort; (2) Polygenic Risk Scores (PRS) were derived from this GWAS for individuals in the Avon Longitudinal Study of Parents and Children (ALSPAC, UK) and used to examine associations with social traits for GWAS validation; (3) the genetic correlation between Social-isolation and psychiatric disorders was examined using GWAS results from the Psychiatric Genomics Consortium (PGC), and (4)). Finally, Mendelian Randomization (MR) was applied to estimate causal effects between Social-isolation and psychiatric disorders.

## Results

### GWAS

**Figure 1:**
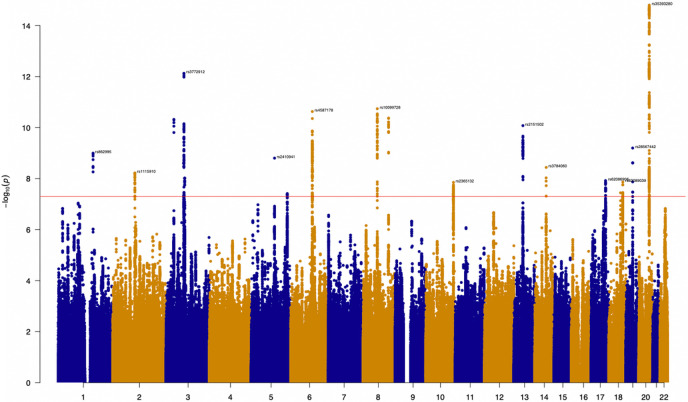
Manhattan plot for Social-isolation after conditioning on psychiatric disorders, based on the meta-analysis of 4 traits. The red horizontal line denotes genome-wide significance (*P* < 5x10^−08^). See **Supplementary Table 5** for details on genome-wide significant SNPs.

To investigate genetic propensity towards social isolation behavior (Social-isolation), a GWAS was performed in the UK Biobank, based on a composite of 4 self-reported behavioral traits pertaining to this behavior. LD Score regression revealed that the individual traits were genetically correlated (see **Supplementary Table 4**). These were meta-analyzed with MTAG before being conditioned on schizophrenia (SCZ), major depressive disorder (MDD), and autism spectrum disorder (ASD), using mtCOJO. The initial GWAS identified 19 loci, post-conditioning 17 loci remained at genome-wide significance (*P* < 5x10^−08^; see Fig. 1).

The majority of the SNPs found to be associated with Social-isolation were not previously associated with psychiatric or neurodevelopmental disorders. However, there are several exceptions. For example, the top lead SNP (rs67777906; *P* = 1.80x10^−15^) is situated in the ARFGEF2 gene, implicated in distinguishing between bipolar disorder (BD) and SCZ^32^, as well as post-traumatic stress disorder (PTSD)^33,34^. The second top SNP in chromosome 8, and the fourth top hit overall (rs2721942; 1.47x10^−10^), has also been associated with Post Traumatic Stress Disorder (PTSD)^35^. In chromosome 19, the lead SNP (rs28567442; *P* = 6.31x10^−10^) is embedded in ZNF536, implicated in the development of the forebrain, and associated with SCZ^36^. Other genome-wide significant SNPs are in genes associated with SCZ (rs6125539; 4.72x10^−09^; CSE1L)^32^ and impulsivity (rs1248860; 9.51x10^−09^; CADM2)^37^. In chromosome 13, rs17057528 (*P* = 8.82x10^−09^) is in DIAPH3, identified as an autism risk gene^38^, and is also implicated in hearing loss and impairment of speech perception^39^.

### Polygenic risk scores

#### ALSPAC

To validate the Social-isolation GWAS and PRS in an independent cohort, as well as explore its generalizability to a developmental cohort, PRS were generated in ALSPAC using the 13 significance thresholds for SNP inclusion. The PRS were used to examine associations with friendship scores, comprising the 5 items relating to peer contact in n = 4,934 (at age 12) and n = 2,909 (at age 18) participants of the ALSPAC cohort.

The Social-isolation PRS were not associated friendship scores at age 12. At age 18, friendship score was significantly associated with the Social-isolation PRS at the *P*_T_ = 0.05 and *P*_T =_ 0.1 threshold, with the latter being the most strongly associated (*r^2^* = 0.006, P = 0.001; see **Supplementary Tables 6 and 7** for full results). The fewer SNPs were included, the less the predictive the model in terms of p-value, with the genome-wide significant only SNPs not associated with the friendships scores. This demonstrates the signal included in SNPs that did not reach genome-wide significance in contributing towards social isolation behavior.

#### LD score regression

LD score regression was performed to investigate genetic correlations between Social-isolation in the UK Biobank and schizophrenia (SCZ), major depressive disorder (MDD) and autism spectrum disorder (ASD) from the Psychiatric Genomics Consortium (PGC). All 3 psychiatric disorders were correlated with Social-isolation, with ASD having the strongest genetic correlation (*rg* = 0.23, SE = 0.048, *P* = 2.25x10^−06^), followed by SCZ (*rg* = 0.102, SE = 0.028, P = 0.0002) and MDD (*rg* = 0.093, SE = 0.035, *P* = 0.009). The results indicate that Social-isolation genetics are associated with the genetics of these psychiatric disorders and may form part of the genetic basis for them. This could occur if the genetics of Social-isolation have downstream effects on behavior that could increase risk of symptoms and eventual diagnosis, or if the diagnosis itself leads to increased Social-isolation. Using LD score regression, the SNP-heritability of Social-isolation after conditioning on psychiatric disorders was estimated to be *h^2^* = 0.04 (SE = 0.0022, P = 8.95x10^−77^), suggesting a small but significant SNP-based heritable component.

#### Mendelian randomization

Using the MR-Egger method, there was no evidence of causal relationships between Social-isolation and psychiatric disorders with the exception of ASD having a causal effect on Social-isolation when SNPs were selected as instruments at the 5x10^−05^ threshold (Beta = 0.019, SE = 0.0052, P = 0.00041). However, MR-Egger is a conservative method and may be underpowered to detect causal associations in complex behavioral traits. See **Supplementary Table 8** for full results.

## Discussion

This is the first study of the genetic factors that contribute to the behavior of social isolation. A GWAS identified 17 genetic loci which predispose towards social isolation behavior. Some of these were in genes previously associated with psychiatric and neurological disorders, as well as neurotransmitter and brain function. However, most were not previously found to be associated with other mental health, neurodevelopmental, or personality traits. Polygenic risk scores (PRS) derived from the GWAS were associated with the friendship scores at age 18 and there was strong evidence supporting shared genetic etiology between Social-isolation and major psychiatric disorders, based on genetic correlations.

The PRS generated in ALSPAC were associated with friendship scores at age 18 but not at age 12. These results suggest Social-isolation GWAS is valid indicator of social-related traits, with higher PRS associated with worse friendship satisfaction and outcomes. The PRS being associated scores at age 18 as opposed to 12 might indicate that genetically influenced personal social behavior does not necessarily manifest until later in adolescence. This finding could be due to confounding by gene-environment correlation^40^. At younger ages, children may have less control over their own social environments and interactions than at age 18, as their parents would likely select their environments for them, in which case behavior would be less strongly influenced by their own genetic predispositions. A similar effect is observed in intelligence genetics, in which heritability increases over time^41^. It is considered that genetic predisposition leads to active and passive correlations with school selection or teacher attention, for example, creating a “snowball” effect in which those genetic influences are amplified over time. It is possible that similar effects are at play with behavioral genetics, in which Social-isolation genetic predisposition lead to development, or lack thereof, of social skills and sociability, modulating social isolation over time.

Social-isolation was found to be genetically correlated with SCZ, as well as with ASD and MDD. This pattern of results suggest that Social-isolation is a feature that cuts across multiple psychiatric disorders and mental health generally. It is well known that social isolation is linked to poorer mental health^42^, but here it is shown that there is a genetic association which indicates that Social-isolation may form part of the aetiological basis of these disorders. Further studies with psychiatric cases will be required to test this hypothesis, but considering that social engagement is an easily modifiable intervention target^43^, identifying those with a genetic predisposition towards Social-isolation may be a useful strategy in mitigating mental health issues.

The current study was able to demonstrate a heritable genetic component to Social-isolation by utilizing a large sample size and detailed phenotype information, allowing a comprehensive and valid Social-isolation trait to be developed. This was confirmed by the PRS generated from the GWAS being validated in an independent sample, and several genome-wide significant SNPs found associated with Social-isolation. However, LD Score Regression only estimated 4% heritability for Social-isolation and PRS were only able to explain 0.6% of the variance in friendship scores in ALSPAC replication sample. The SNP-heritability is likely to be a lower bound estimate, as this only takes into account the common SNPs genotyped and not rare variants or de novo mutations^44^. Further, despite having up to 450,000 individuals available for the GWAS, the most powerful GWAS such as educational attainment are becoming increasingly predictive with approximately 3 million participants^45^. Thus, increasing sample size will allow the detection of more SNPs that contribute to Social-isolation behavior and increase both heritability estimates and the predictive power of PRS. In ALSPAC the target sample also had relatively few participants at age 18 (n = 2,909) compared to age 12 (n = 4,934), which likely contributed to lower bound variance explained.

In order to further investigate how the genetic component of Social-isolation manifests in behavior and the development of psychiatric disorders, further studies will be required which investigate whether or not Social-isolation PRS are able to predict case control status for disorders such as SCZ, MDD and ASD. If so, it will be necessary to consider which specific behaviors are influenced by genetics, and how these manifest in the development and diagnosis of psychiatric disorders. By targeting behavior, our present study has laid the foundation for identifying a possible target for intervention that can be addressed in real world scenarios. However, the relatively small effect sizes of individual SNPs and the resulting low predictive power of PRS mean further investigation is necessary.

## Methods

### Study cohorts

#### Discovery sample

The **UK Biobank (UKB)** is a detailed prospective study with over 502,650 participants aged 40–69 years when recruited in 2006–2010, and includes both genetic and phenotypic data on complex traits^19^. The recruitment process was coordinated around 22 centers in the UK (between 2007 and 2010). Individuals within travelling distance of these centers were identified using NHS patient registers (response rate = 5.47%). Invitations were sent using a stratified approach to ensure demographic parameters were in concordance with the general population. All participants provided written informed consent and the current study was ethically approved by the UK Biobank Ethics and Governance Council (REC reference 11/NW/0382; UK Biobank application reference 18177).

#### Genetic data

Blood samples from 488,366 UK Biobank participants were genotyped using the UK BiLEVE array or the UK Biobank axiom array. Further details on the genotyping and quality control (QC) can be found on the UK Biobank website (http://www.ukbiobank.ac.uk/scientists-3/genetic-data/). In the current study, SNPs were removed if they had missingness < 0.02 and a minor allele frequency (MAF) < 0.01. Exclusions based on heterozygosity and missingness were implemented according to UK Biobank recommendations (http://biobank.ctsu.ox.ac.uk/showcase/label.cgi?id=100314). Samples were removed if they were discordant for sex. SNPs deviating from Hardy-Weinberg equilibrium (HWE) were removed at a threshold of *P* < 1x10^−8^. Genotype data was imputed according to standard UK Biobank procedure, on 487,442 samples^20^, excluding variants with an MAF < 0.01 and an imputation quality score < 0.3. After basic QC procedures and exclusions, 488,337 samples with phenotype data remained for genetic analysis. Excluding those of non-European ancestry using 4-mean clustering on the first two principal components, 449,609 samples remained for genetic analysis.

#### Phenotype data

##### Social isolation:

To derive a comprehensive measure of Social Isolation (Social-isolation), we ran a data-driven principal component analyses (using Promax rotation) on self-reported answers to questions that (1) directly probed for the quantity or quality of social engagement, and (2) were available for at least 90% of study participants. Based on these criteria, we included data on the following 3 items, that all loaded on a single factor: “Frequency of family/friend visits”, “Being able to confide in others”, and “Number of social activities a week”. The items “Frequency of family/friend visits” and “Being able to confide with others” were both rated on a seven-point Likert scale (i.e. ‘Almost daily’, ‘2–4 times a week’, ‘about once a week’, ‘about once a month’, ‘once every few months’, ‘never or almost never’, and ‘no friends/ family outside of household’). The items “Frequency of family/friend visits” and “Being able to confide with others” were considered continuously and recoded so that higher values corresponded to greater social isolation. Answer options for the item “Number of a/social activities a week” included attending a sports club, pub, social club, religious group, adult educational classes, or other group activities and were summed to represent the ‘total number of social activities a week’, also considered continuously.

To complement the answers to the self-report, sociodemographic information about the number of people in the household was added as additional proxy of social contact. This “Number in household” item was dichotomized as a binary trait representing living alone, with 0 others in household coded as ‘1’ for Social-isolation and any greater number in household as ‘0’. See **supplementary material** for full phenotype and coding details. For all items, individuals with missing data, or who preferred not to answer were excluded. Participants who were wheelchair users or morbidly obese (BMI > 40) were also excluded from the analysis, as these factors may arguably hamper the level of social activity, but are unrelated to genetic or psychiatric vulnerability.

### Validation cohort

#### ALSPAC cohort

The Avon Longitudinal Study of Parents and Children (ALSPAC) is a prospective birth cohort which recruited pregnant women with expected delivery dates between April 1991 and December 1992 from Bristol UK. 14,541 pregnant women were initially enrolled with 14,062 children born and 13,988 alive at 1 year of age. Detailed information on health and development of children and their parents were collected from regular clinic visits and completion of questionnaires. Please note that the study website contains details of all the data that is available through a fully searchable data dictionary and variable search tool” and reference the following webpage: http://www.bristol.ac.uk/alspac/researchers/our-data/. A detailed description of the cohort has been previously published^21,22^. Ethical approval for the study was obtained from the ALSPAC Ethics and Law Committee and the Local Research Ethics Committees.

#### Genotype data

9,115 participants in ALSPAC have genotype data available, after individuals with non-European ancestry were removed. ALSPAC children were genotyped using the Illumina HumanHap550 quad chip genotyping platforms. SNPs with a MAF of < 0.01, a call rate of < 0.95 or evidence for violations of Hardy-Weinberg equilibrium (P < 5x10^−07^) were removed. Data was imputed using standard ALSPAC procedure using the HapMap 2 reference panel, keeping SNPs with MAF > 0.02 and an INFO score > 0.9. This resulted in 4,731,235 SNPs in the analysis. Full quality control procedures can be found at: https://alspac.github.io/omics_documentation/alspac_omics_data_catalogue.html

#### Phenotype data

To test the validity of the Social-isolation construct, 2 friendship scores were derived from 5 questions from clinical questionnaires based on questions from the Cambridge Hormones and Moods Project Friendship Questionnaire^23^, completed by the parents of offspring at ages 12 and 18 respectively e.g. *“Teenager is happy with number of friends”.* Each question consisted of 4 to 6 categorical responses, corresponding to a 4 to 6 point scale e.g. “1 = Very happy, 2 = Quite happy, 3 = Quite unhappy, 4 = Unhappy, 5 = No friends”. Responses were summed to create a continuous scale, with higher scores corresponding to lower friendship quality and greater Social-isolation. 4, 934 of the cohort had the phenotype information at age 12, and 2,909 at age 18. See **supplementary table 2** for full details on questions.

#### GWAS summary statistics

To test for genetic correlations between Social-isolation and associated psychiatric disorders using LD score regression, the Social-isolation GWAS based on UK Biobank data was used along 3 base genome-wide association summary statistics for schizophrenia (SCZ), depression (MDD), and autism spectrum disorder (ASD). These were the Psychiatric Genomics Consortium Wave 3 (PGC3) SCZ GWAS^24^, the 2019 PGC MDD Working Group GWAS^25^, and the 2017 PGC ASD Working Group GWAS^26^.

### Statistical analyses

#### GWAS analysis

Association testing of autosomal SNPs was carried out on each of the 4 Social-isolation traits using BOLT Bayesian linear mixed models (BOLT-LMM)^27^ to account for relatedness and cryptic population stratification, while increasing power and controlling for false positives. Age, sex, batch, and center were included as covariates, as well as education, income, and Townsend deprivation index (TDI) to account for socio-economic status (SES). The top 15 principal components (PCs) were also included to control for main population stratification. MTAG^28^ was used to meta-analyze the individual *“Frequency of family/friend visits”*, *“Being able to confide in others”*, *“Number of social activities a week”*, and *“Number in household”* outcomes to form a single, composite Social-isolation GWAS. This score is achieved by leveraging power across correlated GWAS estimates in overlapping samples. Finally, multitrait-based conditional and joint analysis (mtCOJO)^29^ was used to adjust the Social-isolation GWAS summary statistics for the effects of psychiatric disorders, specifically schizophrenia (SCZ), major depressive disorder (MDD), and autism spectrum disorder (ASD), using European ancestry GWAS summary statistics for each. These are the psychiatric disorders which are commonly considered to lead to increased risk of social withdrawal and isolation^2,3,4,7,8^ and were conditioned on to remove potential downstream effects of psychiatric disorders. SNPs were selected as instruments at 5x10^−05^, clumped 1MB apart or with LD *r^2^* < 0.2 based on the 1000 Genomes Project Phase 3 reference panel for independence. mtCOJO uses these SNPs Generalized Summary-data-based Mendelian Randomization (GSMR) to estimate the effect of the exposures (psychiatric disorders) on the outcome (Social-isolation), producing conditioned effect sizes and p-values. Statistically significant independent signals were identified using 1MB clumping and a genome-wide significance threshold of *P* < 5x10^−08^.

#### Polygenic risk score analysis

Polygenic risk scores (PRS) were generated in ALSPAC using PRSice-2^30^, using the Social-isolation GWAS to sum and weight risk alleles for individuals in each cohort. Social-isolation GWAS results were pruned for linkage disequilibrium (LD) using the p-value informed clumping method in PLINK (--clump-p1 1 -- clump-p2 1 --clump-r2 0.1 --clump-kb 250). This method preferentially retains SNPs with the strongest evidence of association and removes SNPs in LD (r2 > 0.1) that show weaker evidence of association within 250Kb windows, based on LD structure from the HRC reference panel. Subsets of SNPs were selected from the results at 13 increasingly liberal P value thresholds (ranging from p < 5x10^−08,^ to p < 0.5). Risk alleles were included and tested to predict outcomes at 13 different significance thresholds, allowing the utilization of the most predictive PRS and threshold. These PRS were tested for associations with the friendship scores in ALSPAC, using linear regression models and including age, sex and 10 PCs as covariates. To account for the multiple testing of 13 PRS thresholds and 2 friendship scores, a Bonferroni correct significance threshold of *P* < 0.002 was used.

#### LD score regression

Genetic correlations and heritability estimates were conducted using LD score regression^31^, to investigate associations between Social-isolation and SCZ, MDD, and ASD, using GWAS summary statistics from the Social-isolation GWAS conducted in the UK Biobank and each psychiatric disorder from the Psychiatric Genomics Consortium (PGC).

#### Mendelian randomization

To test for causality between Social-isolation and psychiatric outcomes, bi-directional Mendelian Randomization was conducted using the package TwoSampleMR (https://github.com/MRCIEU/TwoSampleMR). Instrumental variables for the exposures (both Social-isolation and the psychiatric disorders SCZ, MDD, and ASD) were extracted at genome-wide significance and at p < 5x10^−05^ after strict LD clumping at 10,000kb windows and LD *r^2^* < 0.001 to ensure instruments were independent. Exposure and outcomes were harmonized and MR-Egger was used in the primary analyses to account for horizontal pleiotropy. The inverse variance weighted (IVW) was also used as a less conservative, more powerful approach. To account for multiple testing, a Bonferroni corrected p-value threshold of P < 0.004 was used to ascertain significance.

## Data Availability

UK Biobank data are available through a procedure described at http://www.ukbiobank.ac.uk/using-the-resource/ ALSPAC data access is through a system of managed open access. The steps below highlight how to apply for access to the data included in this paper and all other ALSPAC data. Please read the ALSPAC access policy (http://www.bristol.ac.uk/media-library/sites/alspac/documents/researchers/data-access/ALSPAC_Access_Policy.pdf) which describes the process of accessing the data and biological samples in detail, and outlines the costs associated with doing so. You may also find it useful to browse our fully searchable research proposals database (https://proposals.epi.bristol.ac.uk/), which lists all research projects that have been approved since April 2011. Please submit your research proposal (https://proposals.epi.bristol.ac.uk/) for consideration by the ALSPAC Executive Committee using the online process. You will receive a response within 10 working days to advise you whether your proposal has been approved. If you have any questions about accessing data, please alspac-data@bristol.ac.uk. Schizophrenia, Autism spectrum disorder, and major depressive disorder GWAS summary statistics are publicly available from the PGC (https://www.med.unc.edu/pgc/download-results/)

## Code availability

Software code for *PRSice-2* is available at https://www.prsice.info/. All other code used is available upon request.
